# Mapping mental health complexity in esports: an exploratory psychosocial network approach

**DOI:** 10.1186/s40359-026-05800-y

**Published:** 2026-10-19

**Authors:** Lucas Van Ruysevelt, Martijn Van Heel, Michael G. Trotter, Matthew Watson, Ismael Pedraza-Ramirez, Iván Bonilla, Paul Davis, Jóhann Ragnarsson, Olivier Mairesse, Jolan Kegelaers

**Affiliations:** 1 Faculty of Psychology and Educational Sciences, Vrije Universiteit Brussel, Brussels, Belgium; 2 School of Health and Welfare, Halmstad University, Halmstad, Sweden; 3 Belgian Esports Federation, Brussels, Belgium; 4 Institute of Psychology, German Sports University Cologne, Cologne, Germany; 5 G2 Esports, Berlin, Germany; 6 Department of Basic, Developmental, and Educational Psychology, Universitat Autònoma de Barcelona, Barcelona, Spain; 7 National Institute of Physical Education of Catalonia, Universidad de Lleida, Lleida, Spain; 8 Department of Psychology, Umeå University, Umeå, Sweden; 9 Rafíþróttasamband Íslands, Kópavogur, Iceland

**Keywords:** Bayesian network; Depression; Directed Acyclic Graph; Emotion regulation; Esports; Game preoccupation; Mental fatigue; Mental health; Psychosocial network analysis; Sleep

## Abstract

**Background:**

The rapid rise in popularity of esports has brought increased attention to the unique mental health challenges faced by esports players, yet research in this area remains in its infancy. Early research suggests a substantial mental health burden and has examined associations between esports-specific stressors, mental health, and sleep disturbances. However, much of this work has relied on traditional methodologies, providing limited insight into how affective, psychosocial, sleep-related, and esports-specific indicators are organised within this competitive performance environment.

**Methods:**

To address this gap, we developed a bespoke 17-item questionnaire tailored for Directed Acyclic Graph (DAG) network analysis, an exploratory approach for mapping the conditional dependency structure of complex multivariate systems such as mental health.

**Results:**

A total of 1025 esports players from over 60 countries completed the survey. Game-related thought preoccupation, a core feature of gaming disorder, emerged as the most robust structural sink node, downstream of several psychosocial indicators, raising the hypothesis that it represents a compound indicator of broader upstream psychosocial strain. Depressed mood occupied an upstream position within the network, an observation that held more robustly for the psychopathological subnetwork as a whole than for depressed mood as the single source. Mental fatigue occupied an intermediate, connecting position between affective variables and the sleep and fatigue indicators. Its high prevalence, together with a positioning upstream of the sleep indicators, suggests that it may warrant closer examination in future research on training load, recovery, and sleep in esports, though this ordering rested on edges with moderate directional support. Emotion control and a sense of autonomy appeared to connect upstream affective distress with downstream esports-specific indicators such as esports-related stress, though was the least stable of the structural features identified.

**Conclusions:**

Collectively, these findings provide an exploratory map of how mental health-relevant indicators were organised in this performance environment, and generate concrete, testable hypotheses for future longitudinal, experimental, and intervention research.

**Supplementary Information:**

The online version contains supplementary material available at https://doi.org/10.1186/s40359-026-05800-y.

## Introduction

Esports, involving the casual or organised activity of competitive video gaming, facilitated by in-game ranking systems or organised competitions [1], has grown at an extraordinary pace in recent years. While solidifying itself as a prominent global phenomenon, an increasing number of amateur, semi-professional and professional players have entered a competitive performance environment [2]. Esports, and gaming more broadly, has shown to have beneficial effects on, for example, cognitive flexibility and the social connectedness of players (e.g., 3, 4). However, it is also characterised by long, sedentary training schedules, an anti-social online climate, poor sleep, and high levels of performance pressure (e.g., 5). As the industry continues to grow, so too does the recognition of these performance-related demands and their potential influence on the emotional well-being, social functioning, and mental health of players [6, 7].

Recent evidence indicates a substantial burden of mental health problems and sleep disturbances among esports players [8, 9]. Mental health is often conceptualised through two related but distinct dimensions: positive mental health (i.e., well-being) and mental ill-health (i.e., symptoms of mental disorders such as depression or anxiety) [10]. Guided by this framework, and in line with a handful of smaller scale studies [11, 12], Kegelaers and colleagues [8] most recently found prevalence rates of 44.7% for depression, 33.4% for anxiety, 36.7% for poor well-being, and 69.3% for sleep disturbances in a large sample of 1105 esport players across different performance levels. Similarly, a systematic review by Akan and colleagues [13] reported both poor subjective and objective sleep levels in esports players, which in turn have been associated with decreased performance, diminished emotional regulation and increased risk of mental disorders [14]. Consequently, recent research has sought to examine which esports-specific, emotional, social, and psychological stressors are embedded within broader patterns of player well-being and mental ill-health in competitive gaming contexts [6].

When discussing mental health, it is important to highlight its nature as a complex system rather than a single-cause phenomenon [15]. Subsequently, understanding the aetiology of mental health problems in esports is challenging, as these problems are likely the result of complex, dynamic, and mutually reinforcing interactions among individual, social, environmental, and digital performance factors [16, 17]. Several studies have suggested that esports players face a wide array of esports-specific stressors and health challenges (e.g., performance pressure, anti-social behaviour, intra-team conflicts, balancing life commitments, poor sleeping habits, long sedentary sessions), that may be associated with, or potentially compound, mental health problems [5, 18, 19]. While encountering stressors is an inherent feature of competition, they are likely neither necessary nor sufficient for poor mental health [5, 20]. Prior research has identified key psychosocial characteristics that may exacerbate, mediate, or modulate mental health outcomes among esports players. Esports players commonly report challenges in self-regulation and variable levels of social support (e.g., coaches, teammates, family), which in turn have been linked to lower levels of well-being and potential mental disorders [21, 22, 23]. Furthermore, esports players report varying levels of basic psychological needs satisfaction and thwarting, including autonomy, relatedness, and competence, which have been linked to affect, well-being, and motivation in esports contexts [24, 25, 26]. These issues may be further compounded by socio-emotional difficulties that have been associated with intensive or dysregulated gaming engagement, including emotion dysregulation, subjective feelings of loss of control in life, and an increase in interpersonal conflicts, which may add to the mental health burden experienced by some esports players [27]. Additionally, research suggests that training and coaching structures, or the lack thereof, may contribute to player well-being [17]. In grind-oriented environments with limited professional oversight, players may have fewer opportunities to develop adaptive self-regulation strategies and access consistent, high-quality support [28].

Although research has identified common stressors and demands within the esports environment, as well as distinct psychosocial vulnerabilities of esports players, comparatively little is known about how these factors are organised within competitive gaming as a digitally mediated performance environment [5, 6]. One notable study examined the associations between esports-related stressors and mental ill-health outcomes [19], showing that stressors such as game uncertainty, personal worries, in-game pressure, and sleep quality significantly predicted burnout, anxiety, and depression. Complementing these regression findings, Poulus and colleagues [29] used undirected network analysis to show the structure of partial correlations among resilience factors, coping skills, and burnout symptoms within a sample of esports players emphasising the complexity of, and interaction among, psychological factors. While these studies provide a valuable impetus in the domain of mental health research in esports, they do not address how different esports-related-stressors, sleep disturbances, socio-emotional factors, and mental health outcomes are organised as part of the broader mental health ecology.

Much of the existing literature follows an additive risk-factor logic, in which esport-related stressors, sleep problems, or psychosocial vulnerabilities are examined as separate predictors of mental health outcomes. While such work is highly valuable, the traditional methods of analysis used (i.e., prevalence estimates, correlations, standard regressions, and partial correlations) model outcomes largely one at a time and require a single outcome to be designated in advance, which offers limited insight into how esports-specific concerns relate to the wider system. They cannot readily show whether such concerns are structurally positioned as central features of distress, as downstream correlates of broader psychological strain, or as bridging components connecting affective, regulatory, and/or sleep-related processes. To better capture the structural complexity of mental health within an environment, network approaches such as Bayesian Gaussian Graphical Copula Models (BGGCMs) and Directed Acyclic Graphs (DAGs) should be considered [30, 31].

Together, these advanced statistical models provide a powerful exploratory approach for mapping complex multivariate systems by elucidating patterns of model-derived directed conditional dependency pathways between variables [32]. BGGCMs allow for the estimation of partial correlations between variables while controlling for all others in the network. It offers a nuanced understanding of the structure of mental health symptoms, esports-specific stressors, and other included variables by highlighting which associations remain once shared variance with all other variables is controlled for. DAGs extend this approach by introducing directionality to these relationships, identifying plausible conditionally dependent pathways between variables. Such network approaches offer a promising avenue to generate a constrained set of candidate pathways that can guide future longitudinal, experimental, and intervention-focused research.

The current study applies these network approaches in a primarily exploratory, hypothesis-generating manner [33] to provide insight into the complex, interconnected structure among esports-specific stressors, psychosocial factors (basic psychological needs, social support, emotion regulation), sleep factors, psychosomatic symptoms, and mental ill-health symptoms. Specifically, we aim to elucidate the conditional dependency structure among these variables, generate tentative hypotheses about how they may be structured within the broader psychosocial and performance context of esports, and identify candidate nodes and pathways for future confirmatory, longitudinal, and intervention-focused research. To our knowledge, this is the first study in esports to apply these combined network models, moving beyond bivariate analyses, traditional regressions, and undirected network analyses to provide a more detailed exploratory map of mental health relevant processes in esports.

## Methods

### General analysis routine

The Methods and Results sections follow the reporting standards for psychological network analyses in cross-sectional data as set out by Burger and colleagues [34].

#### Sample and data collection

For this analysis, we included the data collected through a large international cross-sectional online survey. This research was part of the European funded Erasmus+ ‘The Good Game’ project, which aimed to better understand the current state of player well-being and mental health outcomes in esports [8, 35] and investigate complex interactions between esports-specific demands, psychosocial factors, and mental health outcomes. This project was a collaborative effort of industry partners and research institutions from five European countries (i.e., Belgium, Spain, Iceland, Germany, and Sweden). The survey was disseminated through different social media channels of the project partners, as well as through external partnerships with YouTube and Twitch TV channels, with the YouTube partnership being paid. Potential participants could access the online survey through a hyperlink or QR code. No incentives were offered to the participants. As no single definition of who qualifies as an ‘esports player’ is agreed on in the literature [1], we adopted an inclusive operationalisation spanning recreational to elite competition, reflected in five self-reported performance tiers: *recreational* (*“*Occasionally competing online or at the lowest-tier local events”), *foundational* (*“*Regularly competing in lower-tier online competitions, local LAN events, events at the bottom of the regional ladder”), *pre*-*elite* (“Competing in second-tier or regional in-person competitions or online competitions, competing for top-level in-game rankings”), *elite* (*“*Competing in prestigious tournaments with a good number of top-tier teams, slightly below the highest-level international competitions”), and *mastery* level (“Competing in internationally top ranked tournaments, organised by well-established franchises”). The survey was administered in eight languages: English, Dutch, Spanish, Catalan, Icelandic, Swedish, German, and French. Items were translated from the English source version and back-translated, with iterative review by multiple native-speaking members of the project consortium to resolve discrepancies and preserve item meaning across versions. Ethical approval was granted by the Ethical Committee of Human Sciences of the Vrije Universiteit Brussel before distributing the survey. Data collection ran from October 2024 until February 2025, resulting in a sample of 1105 esports players.

#### Survey design/variable selection procedure

Although validated, multi-item questionnaires are well-suited for latent variable modelling, their direct use as nodes in psychological network models can be methodologically problematic [34]. Within traditional reflective scales, items are constructed as partially interchangeable indicators of a common latent construct (e.g., sleep quality, anxiety, depression), reflecting shared variance rather than direct associations between distinct psychological components. This complicates the interpretation of network models, which assume that nodes represent autonomous elements of a system whose connections are substantively interpretable [15, 34]. Consequently, overlapping or redundant items can artificially inflate edge weights and centrality estimates, distorting the network topology and undermining interpretability, emphasising the need for each node to be theoretically justified as a meaningful component of the modelled system. In the present study, we attempted to address this by selecting and constructing items intended to reflect distinct, potentially interacting mental health-relevant symptoms and esports-specific challenges [30, 36, 37].

To this end, we developed a bespoke questionnaire with theoretically grounded single-item indicators driven by conceptual, analytic, and practical considerations: by anchoring each node to a distinct, well-defined construct, we attempted to minimise redundancy-induced network structures, preserve the interpretability of individual nodes, and keep respondent burden manageable, at the cost of construct coverage. The set of constructs were defined first, on the basis of the perspective article by Kegelaers and colleagues [38], to span esports-specific stressors, psychosocial factors, sleep, psychosomatic symptoms, and mental health symptoms. For each construct, a single indicator was formulated to capture its core meaning as directly as possible, prioritising conceptual specificity. Items were identified through literature review and multiple iterative discussions within the project consortium comprising clinical-, sport-, and developmental psychologists, a somnologist, and psychometrician as part of ‘The Good Game’ project, with additional feedback from esports stakeholders (e.g., (e)sports psychologists and other domain experts) and statistical experts with specific expertise in advanced network analysis. Across four rounds of review, each candidate item was assessed for face validity against its intended construct, for conceptual distinctness from neighbouring nodes, and for appropriateness to the esports context. Items were added, revised or replaced where the consortium judged that they overlapped conceptually or failed to capture the target construct. Where a validated measure existed for a construct, the item was adopted from or inspired by it, and where none was available, the item was developed within the consortium through the process described above. As network estimation places different demands on measurement than scale construction within Classical Test Theory framework [36], we treated the use of single, theoretically anchored indicators as a deliberate design choice that prioritised node distinctness and interpretability over measurement breadth and psychometric precision. The implications of this trade-off are addressed in the Limitations.

As symptoms of anxiety, depression, and sleep disturbance are highly prevalent in esports [8], distinct items assessing their core characteristics were included. Depressed mood and anhedonia were adopted from the PHQ-2, and anxiety and uncontrollable worrying from the GAD-2 [39, 40], reflecting core symptoms of major depression and generalised anxiety as defined in the DSM-5 criteria. Sleep quantity and sleep quality items were inspired by components of the Pittsburgh Sleep Quality Index [41]. Given the demanding training schedules and prolonged cognitive load associated with esports participation [18, 22, 42, 43], we included indicators of mental fatigue, physical fatigue, and somatic symptoms (e.g., pain, weakness, numbness). Beyond symptom indicators, we also included esports-related psychological factors that have been linked to a potential strain on mental health in gaming and esports contexts, including esports-related stress [44], gaming-related thought preoccupation [27], and difficulties with emotion regulation under pressure [22, 23]. Additionally, as a lack of social resources is often highlighted as a potential risk factor for poor mental health in performance environments [22, 23], indicators assessing perceived insufficiency of support in both the personal and esports environment were also included. Finally, drawing on self-determination theory [26] and the work of Hong and colleagues [25], we included indicators of basic psychological needs thwarting (autonomy, competence, and relatedness), as a conceptually relevant framework for understanding players’ experiences in performance contexts.

This resulted in a final 17-item questionnaire (see Table 1). All items used 10-point Likert-type response scales (1 - *almost never*; 10 - *constantly*) to ensure sufficient variability [34]. A common reference period of the past two weeks was used to support consistent interpretation across variables and to minimise measurement-related artifacts that could bias network estimation and edge interpretation.

**Table 1:** Questionnaire items

Construct	Question	Item label
Depressed mood	How often have you felt down, depressed or hopeless?	“DepressedMood”
Anhedonia	How often have you had little interest or pleasure in doing things?	“Anhedonia”
Anxious mood	How often have you felt nervous, anxious or on edge?	“Anxiety”
Excessive worrying	How often have you not been able to stop or control worrying?	“Worrying”
Quality of sleep	How often did you feel you did not sleep *well* enough?	“SleepQuality”
Sleep duration	How often did you feel you did not sleep *long* enough?	“SleepQuantity”
Mental fatigue	How often have you felt mentally fatigued?	“MentalFatigue”
Physical fatigue	How often have you felt physically fatigued?	“PhysicalFatigue”
Somatic symptoms	How often have you experienced any pain, weakness, numbness, tingling or any other physical symptoms?	“Pain”
Esports-related stress	How often have you felt stressed because of your esport?	“EsportsStress”
Gaming-related thought preoccupation	How often have there been periods when all you could think about was the moment that you could play video games?	“GamePreoccupation”
Emotion regulation difficulties under pressure	How often have you experienced difficulties controlling your emotions when under pressure?	“LackEmotionControl”
Lack of perceived support in the personal environment	How often have you felt insufficiently supported by people in your personal environment (e.g., parents, partner, friends, etc.)?	“LackSupportPersonal”
Lack of perceived support in the esports environment	How often have you felt insufficiently supported by people in your esports environment (e.g., coaches, teammates, etc.)?	“LackSupportEsport”
Basic psychological needs thwarting autonomy	How often have you experienced a lack of freedom to make your own decisions in your esport?	“LackAutonomy”
Basic psychological needs thwarting competence	How often have you felt that you were not really skilled in your esport?	“LackSkill”
Basic psychological needs thwarting relatedness	How often have you felt you had no real close relationships with other people in your esport?	“LackConnection”

#### Convergent validity of single-item indicators

To provide external validity evidence for the single-item indicators, we drew on the validated multi-item instruments administered to the same participants within the broader ‘The Good Game’-project survey [8]. Six of the 17 nodes had a validated counterpart in this broader dataset: “DepressedMood” and “Anhedonia” (Patient Health Questionnaire-9; PHQ-9; 45), “Anxiety” and “Worrying” (Generalised Anxiety Disorder-7; GAD-7; 46), and “SleepQuality” and “SleepQuantity” (Pittsburgh Sleep Quality Index; PSQI; 47). The remaining 11 esports-specific and psychosocial indicators had no validated multi-item counterpart and could not be examined this way.

For each affective indicator, a corrected criterion score was computed by summing the items of its source instrument with the conceptually corresponding item removed (e.g., “DepressedMood” was correlated with the PHQ-9 total excluding its depressed mood-item). The two sleep indicators were correlated with the global PSQI score. Convergent validity was quantified with Spearman rank-order correlation (two-tailed) with 95% confidence intervals derived through Fisher r-to-z transformation, applying pairwise deletion. These analyses were conducted in IBM SPSS Statistics (version 31).

#### Estimation methods

An initial Bayesian Gaussian Graphical Copula Model (BGGCM) was estimated to identify key partial correlational relationships among variables and assess the robustness of edge inclusion. BGGCMs were chosen as they provide “a principled alternative to various penalised approaches” (48, p. 2) such as traditional Gaussian Graphical Models, while performing equally well as other estimation methods. They account for ordinal and non-Gaussian data by modelling dependencies via a copula, thus avoiding Gaussian assumptions [48]. The estimations of the BGGCM network were conducted using the Birth-Death Markov Chain Monte Carlo (BDMCMC) algorithm in the *bdgraph* function of the *BDgraph* package in R [49].

Second, a Directed Acyclic Graph (DAG) was estimated using the greedy search Hill Climbing algorithm in the *bnlearn* package in R [50]. DAGs extend the undirected approach of BGGCMs by introducing directionality and conditionally dependent pathways between the variables. In line with existing theoretical and applied research, the ordinal Likert scale data of this study has been treated as continuous as to avoid information loss (e.g., 51).

#### Accuracy and stability of edge-estimates

To assess the accuracy of the edge weight estimates in the BGGCM, 100.000 iterations of the estimation were run with a burn-in time of 50.000 [48]. The convergence trace plot was visually inspected to judge chain stability. Comparisons between different *g.prior* values were made, to select a data-driven Bernoulli Prior Probability which best fit the data. The value was eventually set at 0.1 to ensure a sparse and interpretable network. A *p_link* (i.e., posterior edge inclusion probability) threshold was set at 0.85 and the resulting Maximum A Posteriori probabilities were used to further ensure a sparse and confident network. Finally, multiple chains were run on different seeds and compared to each other using the Hamming distance and Jaccard index to ensure stability across different estimations.

To assess accuracy of the edge estimates of the DAG, we conducted the *boot.strength* routine implemented in the *bnlearn* package [50], using non-parametric bootstrapping with 5000 bootstrap samples with the greedy search Hill Climbing algorithm, with 5 restarts and a *perturb* value of 10 to not remain stuck in local optima [52]. Next, we calculated the average network with the *averaged.network* function of the *bnlearn* package. In line with guidelines and previous empirical papers, we removed the weak edges by adopting a bootstrap adjacency (edge inclusion) threshold of 0.85 (i.e., the proportion of bootstrapped networks in which an edge was present between two nodes, irrespective of orientation), and a directional-probability threshold of 0.50 (i.e., the proportion of bootstrapped networks in which, conditional on the edge being present, it pointed from node A to node B), leaving us with solely the most robust edges [31]. Finally, we used the *arc.strength* function of the *bnlearn* package to quantify each edge’s contribution to model fit in the final averaged network as the change in the Bayesian Information Criterion (i.e., a relative measure of a model’s goodness-of-fit; *BIC-g*) when that edge is removed. Using the same averaged network, we compared its BIC-g with that of an empty network to ensure confident network selection [53].

To verify that the single-item nodes were sufficiently distinct to be modelled separately, topological overlap among the nodes was examined using the *goldbricker* function of the *networktools* package, which flags node pairs relating near-identically to all remaining nodes as candidates for redundancy [54]. This procedure evaluates statistical interchangeability among nodes and is not a test of construct validity. For the flagged pairs, two sensitivity analyses were conducted in which the DAG was re-estimated under identical bootstrapping, strength, and direction criteria: first excluding the node shared across the flagged pairs, and the second replacing the flagged indicators with a single composite. In each re-estimated network, directed reachability was used to test whether the principal structural finding, the sink nodes, and the minimal edge cut were preserved.

Three analyses examined the robustness of the DAG’s directional structure. The Markov equivalence class of the averaged network was derived to distinguish compelled edges, whose orientation is shared by all equivalent structures, from reversible edges, whose orientation is not determined by the graph [50]. The network was then re-derived at more conservative directional thresholds (0.60 and 0.70), and the sink, edge-cut, and source roles were re-evaluated using directed reachability. Finally, a non-parametric case bootstrap (200 resamples) repeated the full pipeline within each resample (*boot.strength*, 500 samples; strength > 0.85; direction > 0.50), expressing the stability of each role as the proportion of resamples in which it held.

#### Statistical packages

The analyses have been conducted using R (version 4.4.3; 55). For the network estimation of the BGGCM, the *bdgraph* function in the *BDgraph* package was used [49]. For the DAG network estimation, the *boot.strength* function in the *bnlearn* package was used [50]. The Markov equivalence class of the averaged DAG was derived using the *cpdag* function of the *bnlearn* package [50]. The centrality measures for both models were calculated using the *strength* and *degree* functions of the *igraph* package, and the *arc.strength* function of the *bnlearn* package for the DAG [50, 56]. Additionally, the *estimateNetwork* function of the *bootnet* package was used for estimating predictability through a Relative Importance Network [57]. Topological overlap among nodes was examined using the *goldbricker* function of the *networktools* package [54]. Finally, all networks have been visualised using the *qgraph* package [58].

### Analysis-specific routine

#### Centrality indices

We estimated node-level centrality indices of the BGGCM using the posterior mean adjacency matrix (*selected_g*), posterior edge inclusion probabilities (*p_link*) and the estimated precision matrix (*K_hat*) derived from the *BDgraph* function with the posterior edge inclusion probabilities thresholded at 0.85. Centrality metrics included partial correlations (i.e., partial correlations between nodes), expected influence (i.e., sum of node-level partial correlations; as all retained edges carried positive weights, the signed and absolute sums coincide), and degree (i.e., number of connections of a node).

To quantify how well a node is connected to other nodes in the DAG structure, we investigated arc strength (i.e., the change in model score when an edge is removed, based on their *BIC-g* score), and incoming and outgoing BIC-g contributions (InBIC and OutBIC; i.e., the summed BIC-g change across a node’s incoming and outgoing edges respectively, rather than partial correlations) as centrality measures. Lastly, the DAG was visually inspected for potential minimal edge cuts (i.e., cases in which removing one or two nodes eliminated directed connectivity between parts of the estimated graph, e.g., 59).

#### Predictability

We calculated node predictability and influence from a Relative Importance Network (RIN), in which Lindeman-Merenda-Gold (*lmg*) values represent the proportion of a node’s variance explained by the other nodes [57]. From the resulting directed importance matrix we derived, for each node, in-strength (total variance explained in that node by all others; predictability) and out-strength (the total variance that node explains in others; influence). Additionally, we computed each node’s adjusted R² from its DAG parents only.

## Results

### General analysis routine

#### Final sample size

A total of 1105 participants were initially recruited as part of the broader project reported in the study of Kegelaers and colleagues [8]. For the current analysis, only respondents who fully completed the distinct questionnaire used in this study were retained. Applying listwise deletion for incomplete cases resulted in a final sample of 1025 participants. Participants had a mean age of 27.74 (SD = 7.37) and were required to be at least 18 years old. The sample comprised 845 participants who identified as man (82.4%), 139 who identified as woman (13.6%), 29 who defined their gender in another way (2.8%), and 12 who preferred not to share their gender identity (1.2%). Reported performance levels ranged from *recreational* (*n* = 288), *foundational* (*n* = 292), *pre*-*elite* (*n* = 232), *elite* (*n* = 144), and *mastery* level (*n* = 69). The number of participants per language version is reported in Supplementary Table S1. The five countries with the highest number of participants were Spain (*n* = 217), United States of America (*n* = 210), Germany (*n* = 204), Sweden (*n* = 49), and France (*n* = 46) (Table S2), and the five most popular games within our sample were League of Legends (*n* = 316), DOTA2 (*n* = 80) and Counter-Strike (*n* = 75) (Table S3).

#### Results of convergent validity of single-item indicators

Convergent coefficients for the affective indicators were moderate, ranging from 0.47 to 0.59 (all *p* < .001; Table S4A). These indicate that the single items relate substantially to the broader internalising domain, though discrimination between depression and anxiety was limited, with several items correlating as strongly with the neighbouring affective construct as with their own. This pattern is consistent with the well-documented comorbidity of depression and anxiety, and with the topological-overlap analysis (see Redundancy and Sensitivity Analyses). Both single sleep items correlated moderately with the global PSQI score, with SleepQuality at 0.60 and SleepQuantity at 0.56 (both *p* < .001; Table S4B), supporting their convergent validity against an established sleep measure.

#### Results of the accuracy and stability checks

**Fig. 1:**
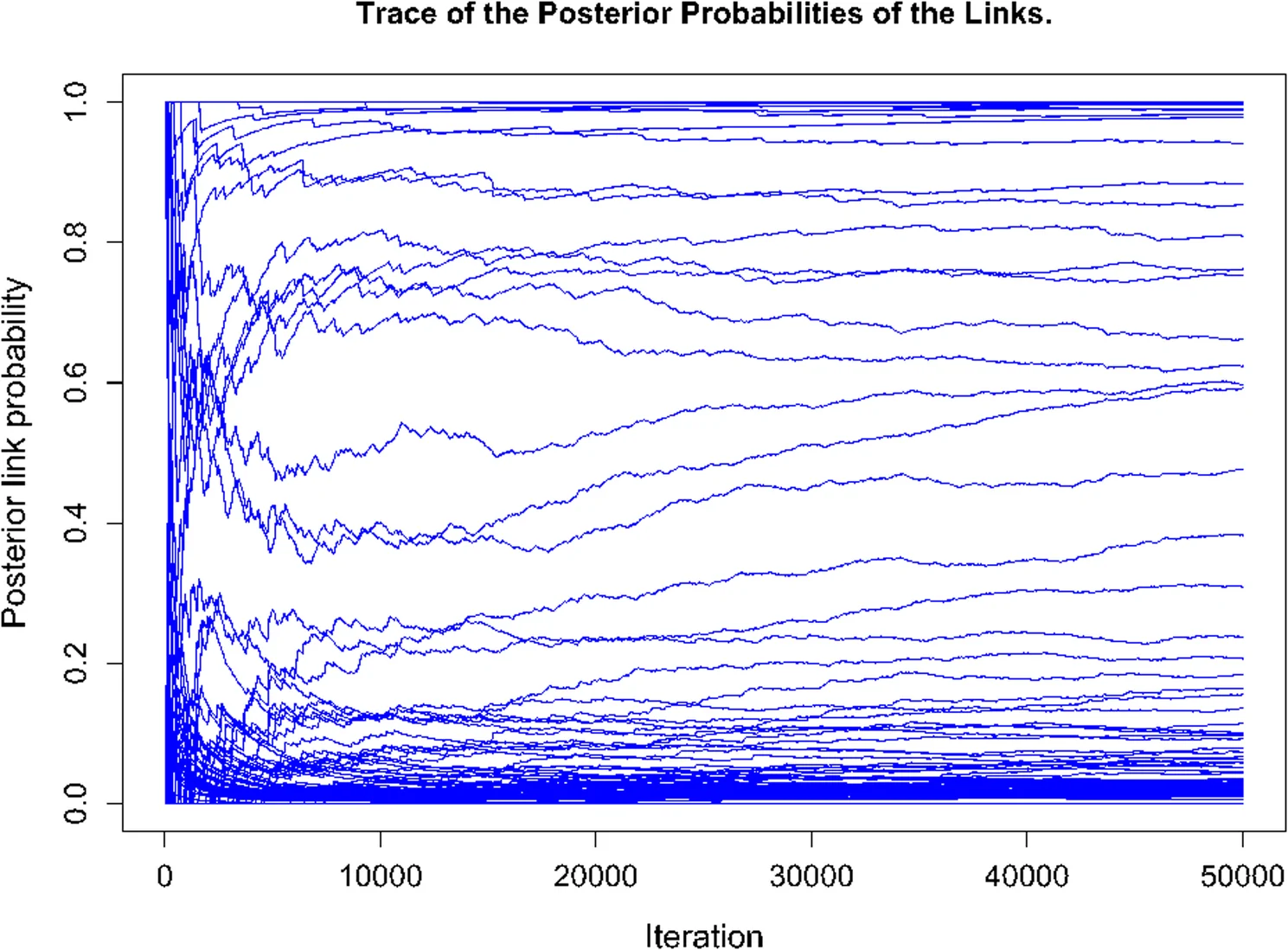
BGGCM Posterior Probabilities convergence plot of all possible links. Description: Convergence plot from the BDgraph plotcoda function. Each line represents the cumulative occupancy fraction (posterior inclusion probability) of a possible edge in the graph across BDMCMC iterations

In estimating the BGGCM, edge probabilities showed strong fluctuations in the initial 5000 bootstrap iterations, but soon stabilised after 10.000 iterations, showing signs of high confidence and convergence (Fig. 1). The calculation of the average normalised Hamming distance and average Jaccard index resulted in excellent respective scores of 0.0098 and 0.9637. Finally, a Posterior Probability (*p_link*) threshold of 0.85 was set [31], resulting in an average absolute edge probability score of 0.9821.

With regards to the DAG estimation, the Hill Climbing algorithm was ran for 5000 bootstrap samples, with five restarts and a perturb value of 10. Respective strength and direction thresholds of 0.85 and 0.5 were set to ensure robust and stable edges. The difference between the model score (i.e., BIC-g) and the score of an empty network was 3691.34, indicating very strong confidence in model selection (53).

### Analysis-specific routine

#### Predictability

Node predictability was estimated from the Relative Importance Network as in-strength: the total variance in a node explained by all others, and complemented by influence, estimated as out-strength: the total variance a node explained in others (Table S5 and S6). “DepressedMood” showed the highest values on both, followed by “Anxiety”, “SleepQuality”, and “PhysicalFatigue”. Esports-specific nodes positioned lowest, with ”GamePreoccupation”, “LackSkill”, and “LackConnection” showing the lowest values across both measures. As a complementary measure restricted to the directed structure, node-level adjusted R² was calculated from each node’s DAG parents (Table S7). “SleepQuality”, “Worrying”, and “Anxiety” and “MentalFatigue” were the best explained by their direct parents, while esports-specific nodes again showed the lowest values.

#### Network visualisation

**Fig. 2:**
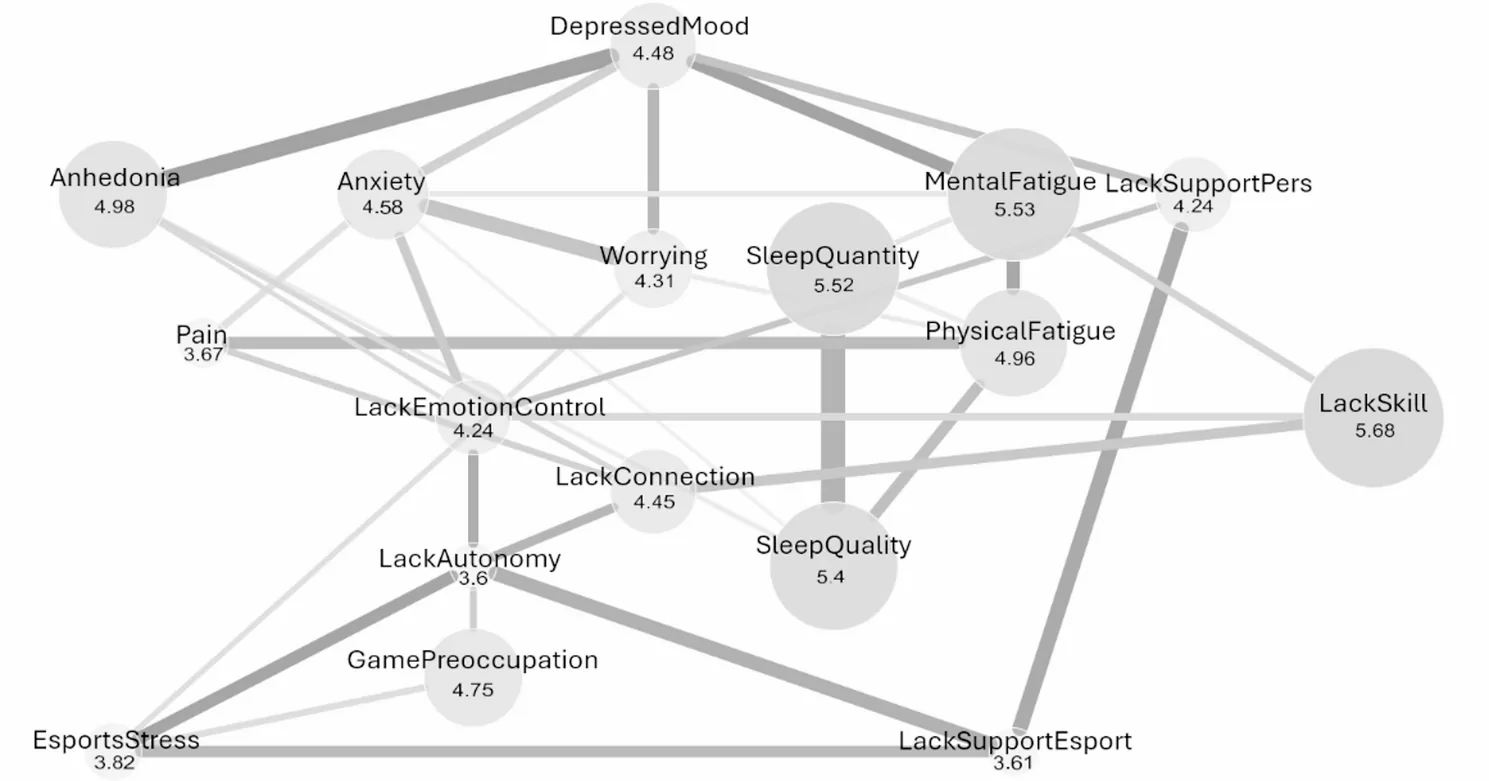
Bayesian Gaussian Graphical Copula Model of the bespoke questionnaire data (edge width based on partial correlations and node size based on mean score). Description: Bayesian Gaussian Graphical Copula Model (BGGCM) network visualisation. Each node represents a questionnaire item, and node size reflects the item mean. Each edge depicts an estimated partial correlation between two nodes, controlling for all other variables in the model; edge presence and thickness reflect the strength of these conditional associations

The BGGCM network visualisation is presented in Fig. 2 and Figure S2. Here, edges represent partial correlations between the copula transformed items. Edge strength (i.e., partial correlation) scores ranged from 0.0735 (“Anxiety” – “SleepQuality”) to 0.4846 (“SleepQuantity” – “SleepQuality”) (see Table S8).

**Fig. 3:**
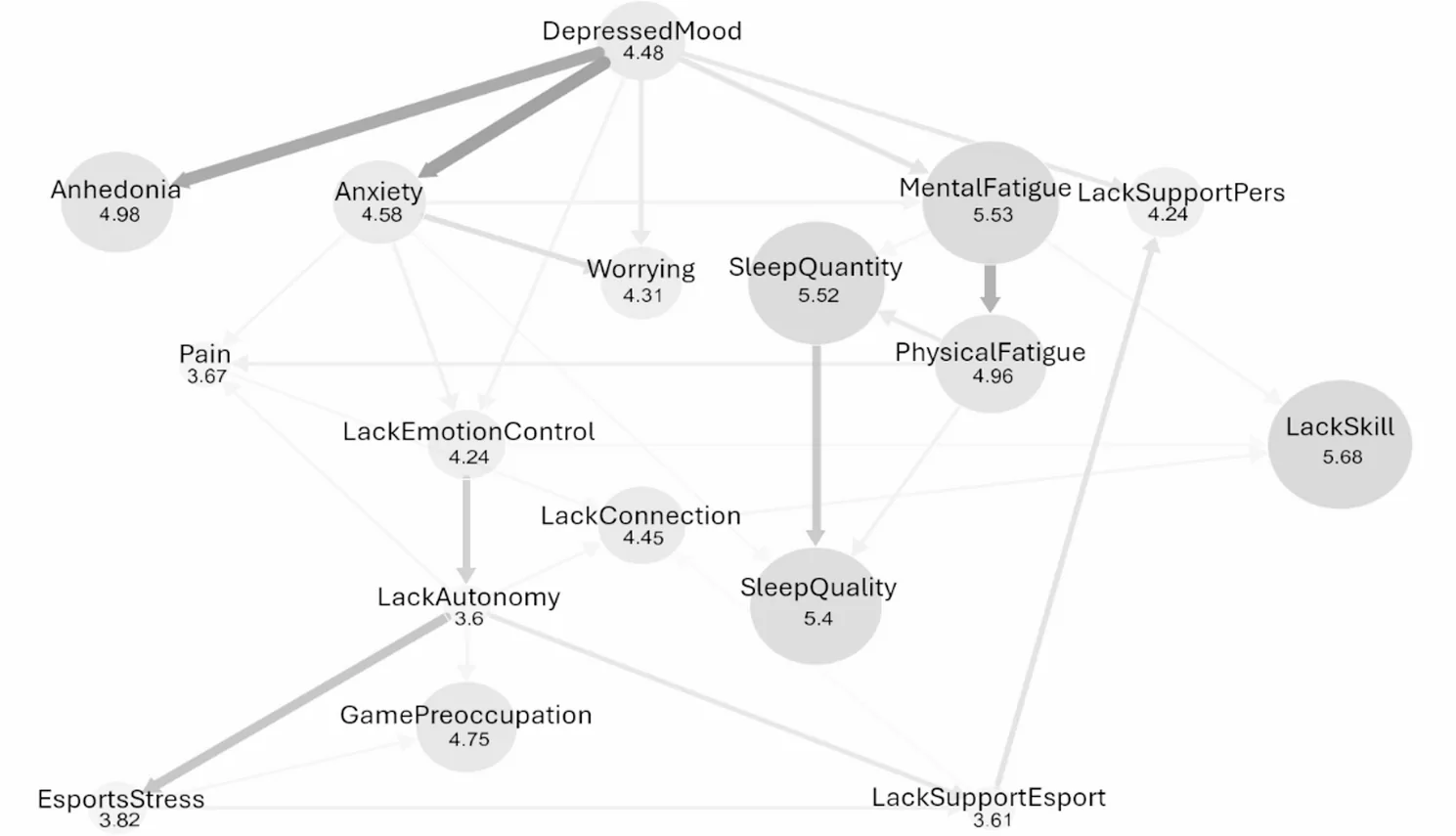
Directed Acyclic Graph of the bespoke questionnaire data – based on BIC-g scores. Description: Directed acyclic graph (DAG) network visualisation. Each node represents a questionnaire item, and its size reflects the item mean. Each directed edge (arrow) depicts an estimated conditional dependency from parent to child in the network; arrow thickness reflects the change in the global Bayesian Information Criterion when that edge is removed from the final averaged network

The DAG network visualisations are presented in Fig. 3 and Figures S3 and S4. In all visualisations, edges that are present in the graph are included because their strength is greater than 0.85 and their direction is greater than 0.50, and represent conditional dependencies between variables (i.e., an arrow from X to Y indicates that Y is modelled conditional on X, with each node conditionally independent of its non-descendants given its parents; 31, 60). It is important to note that the edge direction in the DAG model is model-derived, reflecting statistical conditional dependence rather than definitive evidence of causality or temporal ordering [60]. Moreover, because DAGs impose an acyclic structure, other plausible mechanisms, such as reciprocal or feedback processes, cannot be represented and therefore remain viable alternative interpretations. Edge thickness in Fig. 3 is based on the change in the global Bayesian Information Criterion when that edge is removed from the final averaged network (i.e., the more the edge contributes to the overall model fit, the thicker it is), while edge thickness in Figure S4 represents directional probabilities (i.e., the proportion of bootstrapped networks wherein, conditional on the edge being present, it pointed from node A to node B). Edge weight (*BIC-g* i.e., relative goodness-of-fit measure where more negative values indicate a greater loss in model fit) scores ranged from − 11.26 (from “LackSupportEsport” to “LackConnection”) to -286.12 (from “DepressedMood” to “Anxiety”) (see Table S9). Directional probabilities ranged from 0.52 (from “LackConnection” to “LackSkill”) to 0.97 (from “MentalFatigue” to “LackSkill”) and bootstrap adjacency (edge-inclusion) frequencies ranged from 0.88 (from “Pain” to “LackConnection”) to 1.00 (see Table S10).

To facilitate interpretability of, and consistency between, the BGGCM and DAG visualisation, custom layouts were employed.

#### Network density and average absolute edge weights

To ensure a sparse and interpretable network, a posterior edge inclusion probability (*p_link*) threshold of 0.85 was set in the BGGCM estimations. Based on both the posterior edge inclusion probabilities and Maximum A Posteriori probabilities, this resulted in a total of 34 edges adopted network (25% of 136 total possible edges) with an average absolute edge weight of 0.1908 based on their partial correlations. This percentage of selected edges falls within the ideal boundaries which existing literature describes as an optimal range for psychological data modelling [61].

A bootstrap adjacency threshold of 0.85 (i.e., the edge was present in at least 85% of bootstrap samples) was adopted in the DAG estimation. This resulted in a total of 31 directed edges in the symptom frequency DAG (22.79% of 136 total possible edges), with an average contribution to the model fit of -76.62 based on their Bayesian Information Criterion (*BIC-g*) scores.

#### Centrality indices

Tables S11 and S12 present the results of the centrality analyses of the BGGCM (expected influence, degree). The three most influential nodes based on *Expected Influence* (i.e., the sum of a node’s edge weights) were “DepressedMood,” “LackAutonomy” and “Anxiety.” In contrast, the three least influential nodes were “GamePreoccupation,” “LackSkill” and “Pain.” These BGGCM centrality indices are reported descriptively. We do not interpret the ordering as a stable ranking, and node-level centrality is not the primary inferential target of the study.

Tables S13 and S14 present the results of the centrality analyses of the DAG (InBIC and OutBIC). “DepressedMood” emerged as the only source node of the current network (zero *InBIC*), whereas “Anhedonia,” “GamePreoccupation,” “LackSkill,” “LackSupportPersonal,” “SleepQuality” and “Worrying” emerged as sink nodes (zero *OutBIC*). Furthermore, “DepressedMood” (-812.65), “LackAutonomy” (-341.86) and “MentalFatigue” (-300.48) showed the highest *OutBIC* scores, while “Anxiety” (-286.12), “Anhedonia” (-261.48) and “PhysicalFatigue” (-242.47) showed the highest *InBIC*.

To identify structural bottlenecks in the DAG network, a single-node and two-node cut (or s-t articulation point) analysis was conducted [59]. When these nodes are removed, they interrupt directed reachability within the estimated graph, fully disconnecting one part of the network from another. This analysis showed that “LackEmotionControl” and “LackAutonomy” together formed a minimal edge cut (i.e., removing both nodes eliminated all directed paths from “DepressedMood” and “Anxiety” to “EsportsStress,” “GamePreoccupation” and “LackSupportEsport”). Additionally, the analysis showed that both “MentalFatigue” (eliminating all directed paths from “DepressedMood” and “Anxiety” to “SleepQuantity” and “PhysicalFatigue”), and “DepressedMood” (disconnecting “Anhedonia” from the network if removed) functioned as single minimal edge cut.

#### Specific nodes and edges

The five variables with the highest average score were “LackSkill” (5.68), “MentalFatigue” (5.53), “SleepQuantity” (5.52), “SleepQuality” (5.4) and “Anhedonia” (4.98). In contrast, the five variables with the lowest average score were “LackAutonomy” (3.60), “LackSupportEsport” (3.61), “Pain” (3.67), “EsportsStress” (3.82) and “LackSupportPersonal” (4.24). See Supplementary Table S15 for descriptive statistics of the variables and Supplementary Table S16 for zero-order correlations between variables.

It is important to emphasise that the directionality of the DAG is model-informed, and terms such as source, sink, connecting, upstream, and downstream denote positions in the estimated graph rather than temporal or causal sequence. As such, alternative interpretations, such as reciprocal processes, remain plausible [60]. As we analysed the roles of different nodes in the DAG network, we see that “DepressedMood” appeared as a source node in the estimated graph (*InBIC* = 0.00), with only outgoing and no incoming edges. Conversely, the nodes “Anhedonia,” “GamePreoccupation,” “LackSkill,” “LackSupportPersonal,” “SleepQuality” and “Worrying” functioned as sink nodes (*OutBIC* = 0.00), with only incoming and no outgoing edges.

Based on their BIC-g score, the five edges with the highest contribution to the model fit within the DAG were those from “DepressedMood” to “Anxiety” (-286.12), from “DepressedMood” to “Anhedonia” (-261.48), from “MentalFatigue” to “PhysicalFatigue” (-242.47), from “LackAutonomy” to “EsportsStress” (-178.70), and from “SleepQuantity” to “SleepQuality” (-168.16). These edges represent the best-supported conditional dependencies within the network, with their BIC-g scores indicating the extent to which the model fit deteriorates when the respective edge is removed. In contrast, the five weakest edge contributions to model fit were those from “LackSupportEsport” to “LackConnection” (-11.26), from “LackEmotionControl” to “LackSkill” (-14.40), from “Anxiety” to “SleepQuality” (-16.27), from “EsportsStress” to “GamePreoccupation” (-21.26), and from “Pain” to “LackConnection” (-22.23). While these edges had the weakest BIC-g scores, they were nevertheless present in 85% of the bootstrapped estimations (i.e., although they contribute relatively little to the overall model fit, their presence is consistently supported across resamples). Adjacencies were generally well supported, with almost all edges exceeding a bootstrap adjacency frequency of 0.90, indicating that the presence of these connections was highly stable (Table S10). Across the 31 retained edges, directional support varied considerably (Table S10). Ten edges were well directed (directional probability ≥ 0.80), sixteen showed moderate support (0.60 to 0.79), and five were only weakly directed (< 0.60). The latter group included the edges from “DepressedMood” to “MentalFatigue” (0.58), from “EsportsStress” to “LackSupportEsport” (0.57), from “DepressedMood” to “Anxiety” (0.55), from “Anxiety” to “MentalFatigue” (0.53), and from “LackConnection” to “LackSkill” (0.52). Because the source, sink, and upstream or downstream roles interpreted below depend on edge orientation rather than edge presence, interpretations resting on weakly directed edges are treated as tentative throughout, and their orientations may equally reflect reciprocal or bidirectional relationships that the acyclic model cannot represent.

Visual inspection of the DAG revealed three thematically coherent subnetworks: a psychopathological cluster, a somnological fatigue cluster and an esports-specific cluster. Although these subnetworks remain part of the larger interconnected model, identifying them provides a useful lens for understanding domain-specific processes within the broader network.

**Fig. 4:**
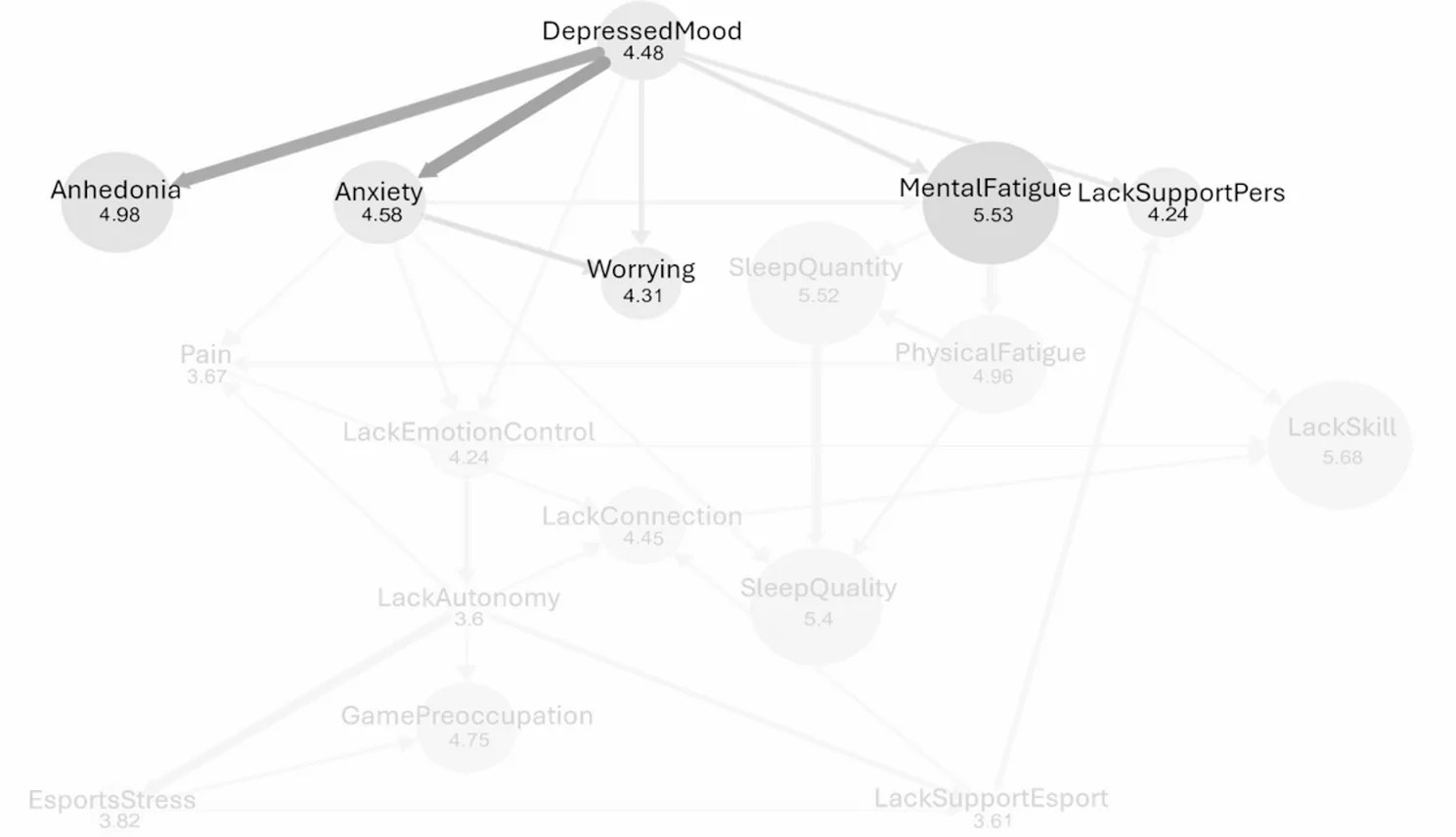
Psychopathological subnetwork – edge thickness based on BIC-g score. Description: Psychopathological subnetwork visualisation. Each node represents a questionnaire item, and node size reflects the item mean. Each directed edge (arrow) depicts an estimated conditional dependency from parent to child within this portion of the network; arrow thickness reflects the change in the global Bayesian Information Criterion when that edge is removed from the final averaged network. Values refer to the full network rather than to the subnetwork estimated in isolation: this psychopathological subnetwork remains part of the larger model but provides a focused lens on domain-specific processes within the broader network

The psychopathological subnetwork (Fig. 4) contains the four variables taken from the PHQ2 and GAD2 questionnaires (“DepressedMood”, “Anhedonia”, “Anxiety” and “Worrying” [39, 40]) alongside “MentalFatigue”, “LackEmotionControl” and “LackSupportPersonal”. “DepressedMood” appeared as a source node. Its edges to “Anhedonia, “LackSupportPersonal” and “LackEmotionControl” were well directed, its edges to “Worrying” showed moderate directional support, and its edges to “MentalFatigue” and “Anxiety” were only weakly directed (Table S10). The edges to “Anhedonia” and “Anxiety” showed the largest BIC-g scores, indicating they were the most influential edges for the network structure, although the orientation of the “DepressedMood”-to-“Anxiety” edge was near-equivocal (Tables S9 and S10).

**Fig. 5:**
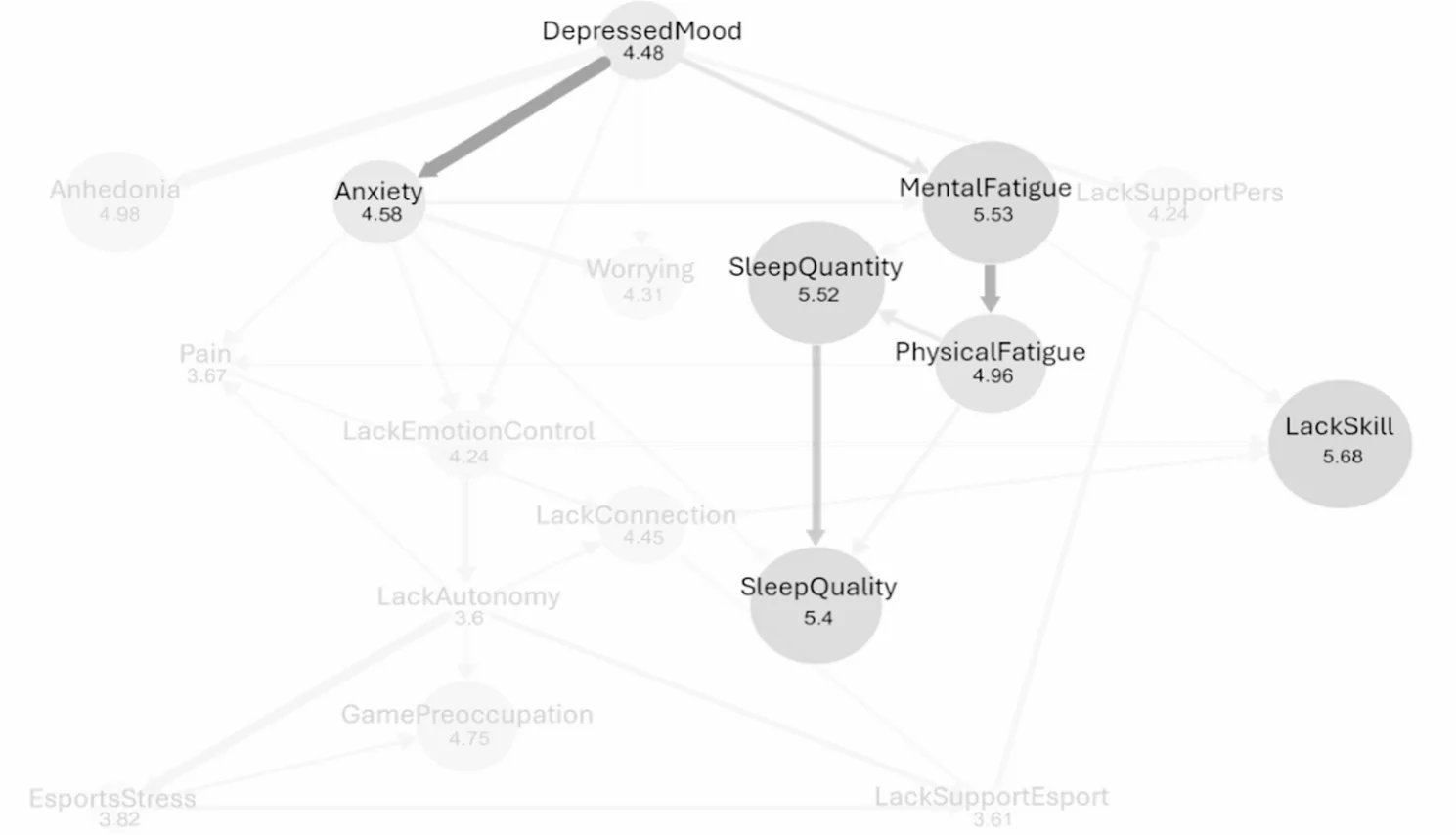
Somnological subnetwork – edge thickness based on BIC-g score. Description: Somnological subnetwork visualisation. Each node represents a questionnaire item, and node size reflects the item mean. Each directed edge (arrow) depicts an estimated conditional dependency from parent to child within this portion of the network; arrow thickness reflects the change in the global Bayesian Information Criterion when that edge is removed from the final averaged network. Values refer to the full network rather than to the subnetwork estimated in isolation: this somnological subnetwork remains part of the larger model but provides a focused lens on domain-specific processes within the broader network

The somnological subnetwork (see Fig. 5) shows the interplay between the sleep quality and quantity variables, physical and mental fatigue variables, perceived lack of skill and affective depression and anxiety variables. The variable “DepressedMood” remained positioned as a source node, with outgoing, weakly directed edges to “MentalFatigue” and “Anxiety”, and through them, indirectly connecting to “SleepQuantity” (from “MentalFatigue”), “SleepQuality” (from “Anxiety”), “PhysicalFatigue” (from “MentalFatigue”), and “LackSkill” (from “MentalFatigue”) (Tables S9 and S10). While the edge from “MentalFatigue” to “LackSkill” had a relatively weak contribution to model fit (BIC-g = -28.94), it had the highest direction of all edges present in the network (dir = 0.97). Additionally, “SleepQuality” functioned as a structural sink node in the network (*OutBIC* = 0.00, *InBIC* = -223.85) and received a strong, moderately directed edge from “SleepQuantity”, a moderate, well directed edge from “PhysicalFatigue” and a weak, moderately directed edge from “Anxiety” (Tables S9 and S10). As mentioned before, the mean scores of “SleepQuality” (5.4), “SleepQuantity” (5.52) and “MentalFatigue” (5.53) were among the highest of the network, marking them as salient problems.

**Fig. 6:**
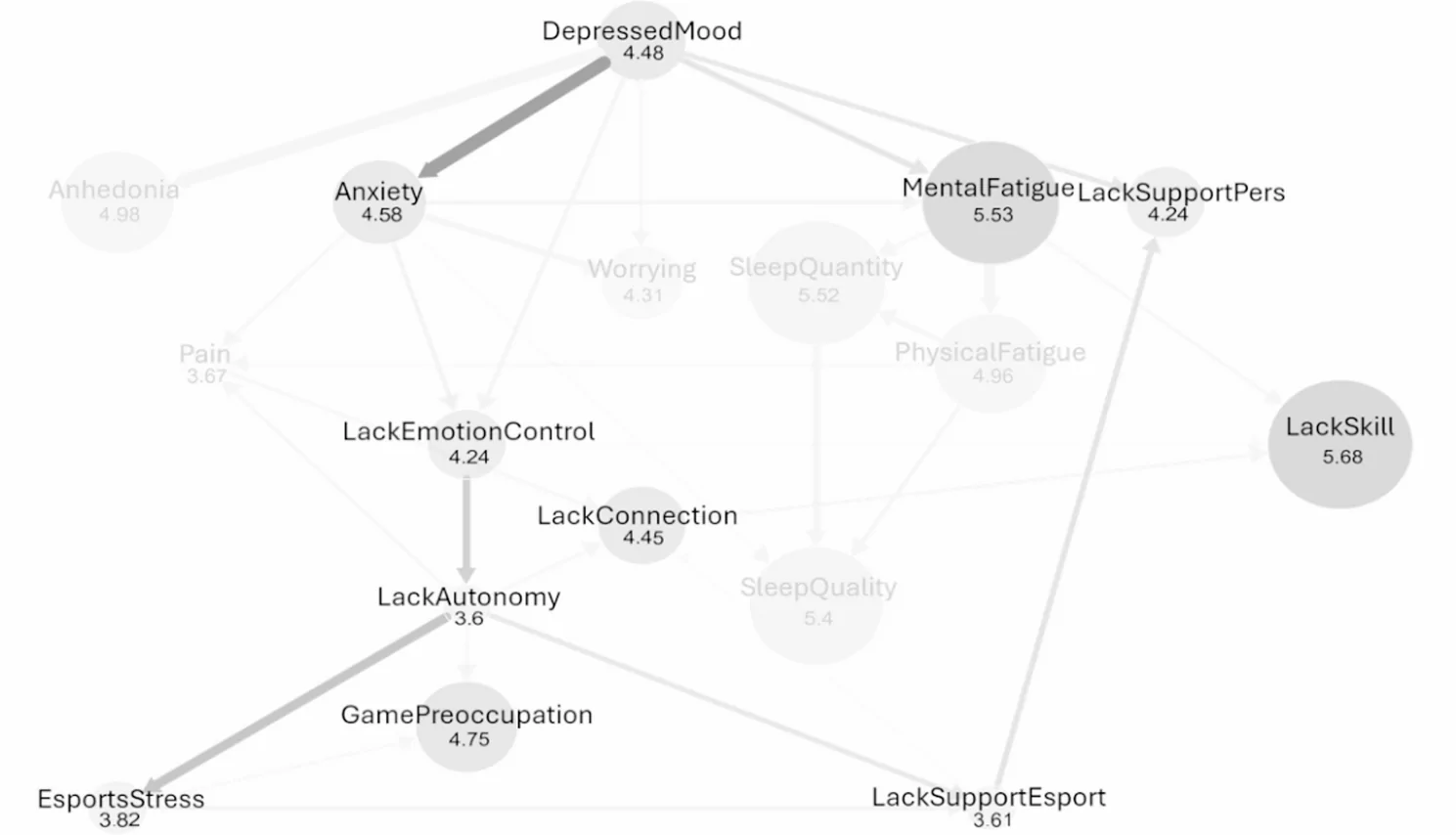
Esports-specific subnetwork – edge thickness based on BIC-g score. Description: Esports-specific subnetwork visualisation. Each node represents a questionnaire item, and node size reflects the item mean. Each directed edge (arrow) depicts an estimated conditional dependency from parent to child within this portion of the network; arrow thickness reflects the change in the global Bayesian Information Criterion when that edge is removed from the final averaged network. Values refer to the full network rather than to the subnetwork estimated in isolation: this esports-specific subnetwork remains part of the larger model but provides a focused lens on domain-specific processes within the broader network

The final thematic cluster is the esports-specific subnetwork (see Fig. 6). Within this cluster, “DepressedMood” again appeared as a source-positioned variable in the estimated DAG, with directed edges to “Anxiety”, “LackEmotionControl”, “LackSupportPersonal” and “MentalFatigue” (Tables S9 and S10). “GamePreoccupation” functioned as the most robust structural sink in the estimated graph (*OutBIC* = 0.00, *InBIC* = -52.97). In addition, “LackEmotionControl” and “LackAutonomy” together formed a minimal edge cut (i.e., removing both nodes eliminated all directed paths from “DepressedMood” and “Anxiety” to “EsportsStress,” “GamePreoccupation” and “LackSupportEsport”), marking them as a candidate structural bottleneck within the current estimated network.

#### Redundancy and sensitivity analyses

The topological-overlap analysis flagged two node pairs within the affective psychopathological cluster as candidates for redundancy (“DepressedMood” and “Anxiety”; “Worrying” and “Anxiety”), consistent with the well-documented co-occurrence of these internalising symptoms. No other pairs were flagged, in particular, the broader single-item constructs (“LackEmotionControl”, “LackAutonomy”, and the support indicators) showed no redundancy, indicating that they occupied statistically distinct positions relative to the remainder of the network. Because this analysis speaks to topological distinctness alone, it does not establish the construct validity of these broader indicators (see Limitations). Additionally, two sensitivity analyses were conducted. Excluding the shared node (“Anxiety”) and re-estimating preserved all principal findings: “GamePreoccupation” and “SleepQuality” remained as structural sink nodes, and “LackEmotionControl” together with “LackAutonomy” continued to form the minimal edge cut. Replacing the three affective indicators with a single composite likewise preserved both sink nodes, however, the minimal edge cut no longer held, as aggregating the affective symptoms opened alternative directed pathways to the esports-specific cluster, bypassing the two cut nodes. Both sink nodes were therefore robust to exclusion and to aggregation of the redundant affective indicators, whereas the bottleneck role of emotion control and autonomy was robust to exclusion of the redundant node but sensitive to coarse aggregation of the affective cluster.

Of the 31 edges, 14 were compelled and 17 reversible within the network’s Markov equivalence class (Table S10). Compelled edges were concentrated on convergent, multi-parent nodes (e.g., “Pain”, “LackConnection”, “SleepQuality”), whereas edges defining the source, sink, and pathway roles were largely reversible, indicating that these orientations, though stable across resamples, are not uniquely determined by the data. Under stricter thresholds, the “GamePreoccupation” sink held at both 0.60 and 0.70, the “SleepQuality” sink and the “LackEmotionControl”-“LackAutonomy” edge cut held at 0.60 but not 0.70, and the sole-source role of “DepressedMood” was lost at 0.60. At 0.70 only 13 edges remained and the network fragmented into seven sources, however, this role loss reflects edge removal more so than reorientation. The case bootstrap showed a similar ordering: the “GamePreoccupation” sink was most stable (88% of resamples), followed by the source role of “DepressedMood” (59%) and the “SleepQuality” sink (54%), while the edge cut was least stable (29%). The “GamePreoccupation” sink is thus the most robust feature of the network, whereas the source role of “DepressedMood” and the strict “LackEmotionControl”-“LackAutonomy” bottleneck are considerably less stable.

## Discussion

As esports has risen in popularity and scale, so too have concerns about the mental health status of players within this competitive gaming environment. With players reporting high levels of mental ill-health outcomes (e.g., depression, anxiety, and sleep disturbances), researchers have questioned which esports-specific factors create a context that may be implicated in these outcomes (e.g., 6, 8, 19). To this end, the aim of this study was to explore the complex interactions between esports-related-stressors, sleep disturbances, socio-emotional factors, and mental health outcomes. We therefore estimated two complementary network models (i.e., BGGCM and DAG), using them in an explicitly exploratory, hypothesis-generating manner [33] to provide description of conditional dependencies among the included variables and generate tentative hypotheses about how these indicators may cluster and relate within a broader competitive gaming ecology [15, 32, 60]. One overarching caution frames all the interpretations that follow. (i) The directionality of the DAG is model-informed, reflecting statistical conditional dependence (i.e., an arrow from X to Y indicates that Y is modelled conditionally on X, with each node conditionally independent of its non-descendants given its parents; 31, 60) rather than causal or temporal antecedence; (ii) terms such as source, sink, connecting, upstream, and downstream denote positions in the estimated graph, not temporal or causal sequence; (iii) the models describe a between-person structure estimated from cross-sectional data and do not necessarily correspond to within-person processes that unfold over time; and (iv) being acyclic, the DAG cannot directly represent the reciprocal or feedback relationships that are plausible for many of these constructs. As such, every structural role and pathway discussed below is offered as a hypothesis to be tested in longitudinal, intervention, or experimental designs.

The psychopathological subnetwork as a whole occupied a notable upstream position within the estimated DAG. Within this subnetwork, depressed mood occupied a source position, sending conditional dependency edges to other variables without receiving any incoming edges, and it was the only source node in the estimated network. Additionally, it was the most upstream node for all three thematic subnetworks (psychopathological, somnological and esports-specific), with edges to anhedonia, anxiety, a lack of emotion control, worrying, mental fatigue and a lack of perceived social support in the personal environment. Its strongest model-fit connections were to anxiety and anhedonia, consistent with the well-known co-occurrence of these mental ill-health symptoms in general, traditional sports, and esports populations [8, 62, 63]. This position was moderately stable rather than definitive as several of its outgoing edges were well directed across bootstraps (i.e., to anhedonia, lack of support in the personal environment and lack of emotion control), but its edges to anxiety and mental fatigue were only weakly directed. We should therefore consider the possibility that these particular relationships are reciprocal or bidirectional. The upstream prominence of the psychopathological subnetwork as a whole is more robust than the specific designation of depressed mood as its single source. Taken together, this indicates that these structures occupied a prominent position in the organisation of the model and tended to co-occur with a wide range of downstream indicators, a pattern reinforced by depressed mood and the other affective variables showing some of the highest out-strength in the Relative Importance Network (RIN). One hypothesis this raises is that low mood plays a structurally similar role across performance domains, consistent with research in traditional sports showing that depressive symptoms are associated with impaired functioning, injury risk, and burnout in elite athletes [64].

A lack of emotion control and a lack of autonomy together formed a bottleneck in the current network structure, positioned between the psychopathological and esports-specific clusters. This role should be read with particular caution, as both are single-item proxies for broad, multidimensional constructs, and their role as the sole bottleneck held only when the affective symptoms were modelled as distinct nodes. Aggregating them opened alternative pathways to the esports-specific cluster, though the two nodes continued to connect the two clusters. Read as a hypothesis, this positioning suggests that emotion control and autonomy may be points at which core psychopathological symptoms and esports-specific outcomes converge. This interpretation aligns with Self-Determination Theory, where autonomy is viewed as central to well-being [65], and with prior findings linking psychological need thwarting to burnout and potential mental health issues in esports [24, 25]. Similarly, difficulties in emotion regulation are known challenges in esports [21, 22, 23], which raises the hypothesis that these difficulties contribute to distinct downstream esports-specific outcomes. Recent syntheses of esports psychology, early intervention work, and diary research likewise highlight the role of stress, coping, and emotion regulation in the well-being of players [17, 66, 67]. The present findings extend this emerging literature and generate more specific hypotheses about how autonomy and emotion regulation link distress to esports-specific outcomes such as esports stress or game-related though preoccupation, for longitudinal and intervention research.

As mentioned, our network revealed a distinct esports-specific cluster downstream of the emotion control and autonomy variables. Within the estimated graph, game-related thought preoccupation occupied a comparatively robust sink position, with only well directed incoming edges and no outgoing edges. One hypothesis it raises is that game-related thought preoccupation may, in some players, function as a compound indicator of broader upstream psychosocial strain rather than an upstream feature itself. This invites future research to consider whether it reflects a maladaptive coping response to mental health difficulties rather than high gaming engagement alone. This interpretation is consistent with research by Bányai and colleagues [68, 69], who found that psychiatric distress was associated with gaming disorder symptoms partly through escapism and coping-related mechanisms. A similar interpretation has been proposed in traditional sports, where compulsive exercise and overtraining have been conceptualised as maladaptive responses to stress and dysregulated affect, rather than primary causes of psychopathology (e.g., 70, 71).

The somnological subnetwork highlights the pervasive sleep-related issues faced by esports players [8, 13, 19]. These indicators were among the most frequently reported symptoms in our sample. In the subnetwork structure, depressed mood remained the most upstream node, connecting to mental fatigue and anxiety, which were in turn connected to perceived lack of skill, sleep quality, sleep quantity, and physical fatigue, with mental fatigue connecting the affective nodes and the sleep and fatigue indicators. Notably, the fatigue-related indicators were positioned upstream of the sleep indicators rather than the reverse. This deviates from conventional clinical framing, in which fatigue is treated as conditionally dependent on poor sleep [72]. This structure raises the hypothesis that poor sleep represents a compound of negative mood and mental fatigue, rather than functioning as an independent upstream element of the wider network [73]. This ordering, however, rested on edges with only moderate to weak directional support (see Table S10), so these relationships may well be bidirectional or reciprocal, and is best read as a tentative observation to be tested directly in longitudinal or intensive-sampling designs.

One hypothesis this raises is that, in high-stress competitive environments, individuals are often thoroughly fatigued yet too cognitively or physically aroused to sleep well [74]. For example, in high-level elite sports, extreme loads, overtraining and insufficient rest are speculated to provoke insomnia-like symptoms (e.g., difficulty falling asleep, restlessness, unrefreshing sleep) despite the athlete’s mental and physical exhaustion [75]. In esports, this may stem from the intense and irregular training regimen characteristic of this environment [42]. Professional, aspiring, and recreational players often engage in prolonged practice sessions, sometimes exceeding 12 h of predominantly sedentary, screen-focused activity, and often occurs in the absence of structured coaching frameworks or evidence-based training methodologies [17, 28, 76]. Moreover, it is not uncommon for esports players to play late into the night, maintaining an irregular sleep schedule and poor sleep hygiene [42]. Such a gruelling schedule may result in significant mental and physical exhaustion, potentially mirroring the impact of high training demands of traditional sports [12]. However, unlike many traditional sports athletes who are educated early about the importance of rest [77], esports players operate in a culture that valorises “grinding” (i.e., excessive practice with minimal breaks), often at the expense of other health considerations such as physical activity or sleep [42, 78. This pattern of insufficient and misaligned sleep, combined with sustained late-night arousal from prolonged screen exposure and competitive stress, may create a scenario in which players end the day thoroughly fatigued but yet physiologically and psychologically aroused, making it difficult to obtain quality rest [42, 79]. As such, our current network generates several directions for future research to examine the role of mental fatigue in esports, including whether it precedes sleep disturbance and whether this association is shaped by mismanaged training loads, late-night play, and pre-sleep arousal.

### Applied implications/future directions

The present DAG findings should be interpreted as exploratory and the directed associations, therefore, form a provisional map of candidate nodes and pathways that may warrant closer attention in future longitudinal, experimental, or intervention studies. Three observations may be especially useful for follow-up work. First, mental ill-health symptoms seem to be both prevalent and structurally prominent in the current network structure, consistent with emerging research (e.g., 8, 9). Together with game thought-preoccupation emerging as a structural sink node, this raises a question future research should address directly: whether players with existing distress are drawn to esports for social, identity-related, or coping functions (i.e., esports-as-coping), whether distress emerges partly through sustained participation in demanding competitive gaming environments (i.e., esports-as-stress), or whether both processes unfold reciprocally over time (e.g. 5, 62, 63, 80). Second, emotion regulation and autonomy occupied a connecting position between distress and esports-specific outcomes that, though less stable than other roles in the network, may merit close examination in future studies of psychosocial functioning in esports, including whether they shape how players experience esports-specific stress and game-related thought preoccupation. Unlike traditional sport, where athletes typically develop within established coaching and team structures, many esports players progress through largely self-directed online play with limited adult guidance. This raises the hypothesis that developing in comparative isolation offers fewer structured opportunities to build emotion regulation skills and a stable sense of autonomy. Future work would benefit from exploring this qualitatively across the course of players’ careers. Third, the high prevalence and positioning of mental fatigue suggest that finer-grained measurement of cognitive fatigue, and its connection to sleep and training load, may warrant attention in future work.

### Limitations

While this study provides a novel and comprehensive look at the interplay between esports-specific stressors, mental health outcomes, socio-emotional factors, and sleep disturbances, it is not without limitations. Firstly, the data are cross-sectional in nature, meaning we cannot definitively establish causal relationships or temporal antecedence [60]. Although the DAG approach introduces directionality based on conditional dependence and model fit, these directions are model-derived and should not be read as causal or temporal claims. As DAGs are acyclic, reciprocal or feedback processes cannot be represented even when they are plausible. A node located downstream in the current topology, such as game-related thought preoccupation, may still play a causal or reinforcing role in the development of mental ill-health symptoms, since its sink position describes between-person conditional dependence rather than the direction of any within-person processes [30]. Edges with directional probabilities near 0.50 may be prone to flipping, so orientations resting on them are interpreted only tentatively. The depressed mood to anxiety edge (dir. = 0.55) is the clearest instance: its near-equivocal orientation is consistent with a reciprocal relationship between these symptoms, which the acyclic model cannot represent.

While we attempted to develop a comprehensive questionnaire, unmeasured variables which are not included in the graph may have a mediating effect and influence the structure of the graph. The measures adopted in the network, although grounded in theory and informed by prior research, were single items for each construct (i.e., one item for autonomy, one for emotion control, etc.). While this choice was intentional, it does mean that each node is a simplified representation of a much more complex and nuanced phenomenon. Furthermore, this simplification is not uniform across the network. Some nodes correspond relatively circumscribed symptoms (e.g., anhedonia, depressed mood), for which a single item captures much of the intended content, whereas others stand in for broader, more multidimensional constructs (e.g., emotion regulation, autonomy) that are ordinarily assessed with multi-item scales. The structural roles of these nodes should accordingly be interpreted with greater caution than those of the narrower symptom nodes. Formal psychometric validation was only partially possible within the present data. Convergent evidence was available for the affective and sleep indicators against established measures (see Results section), but no validated comparator was collected for the broader indicators (emotion control, autonomy, competence, relatedness, and perceived support). These broader nodes should therefore be read as proxies for their target constructs rather than validated measures. The current study did not conduct cognitive interviewing, formal content validity rating, or test-retest assessment, and we did not assess cross-language or cross-group measurement invariance. Establishing these properties remains the task of future dedicated instrument-validation work.

Although the DAG structure may suggest candidate priorities for follow-up work, the present study cannot determine how intervening on any specific node would affect the rest of the network. Our sample was highly heterogenous, consisting of players of all genders (men, women, and nonbinary), ages, performance levels (i.e., recreational to mastery level), and differing geographic regions, and no formal cultural adaptation of item content was undertaken. As measurement invariance was not established, the pooled network should be read as an aggregate across language and cultural contexts rather than as an invariant structure, and as a description of this sample rather than as a universal model of esports mental health. While outside the scope of the current study, formal comparative analyses, and ideally, longitudinal, experimental, and intervention designs are needed before any group-specific or practice-oriented conclusions can be drawn.

## Conclusion

The current study explored the complex, interconnected network structure between esports-specific stressors, psychosocial factors, sleep and fatigue variables and mental ill-health symptoms within a broad esports sample. By combining BGGCM and DAG estimations, we moved beyond prevalences and undirected associations towards an exploratory description of conditional dependency structures, thereby generating hypotheses and suggesting future avenues for longitudinal, experimental, and intervention research. In the network structure, depressed mood occupied an upstream position, an observation that held more robustly for the psychopathological subnetwork as a whole than for depressed mood as the single source. Mental fatigue occupied an intermediate, moderately stable position between negative affect and the broader sleep and fatigue cluster. Emotion control and autonomy together formed a possible connecting structure between upstream affective variables and downstream esports-specific variables, though it was the least stable of the roles identified. Game-related thought preoccupation emerged as the most robust structural sink, downstream of several psychosocial indicators, raising the hypothesis that it represents a compound indicator of upstream psychosocial strain rather than an upstream indicator itself. Taken together, the current findings provide a structured, exploratory map of how a set of theoretically selected indicators was organised in a large sample of esports players and identify specific constructs and pathways that are worth examining more closely in future validation, longitudinal, experimental, and intervention research.

## Supplementary information

Below is the link to the electronic supplementary material.

Supplementary Material 1

## Data Availability

Data will be stored in a public repository upon the completion of the project. Data will be available upon reasonable request.
