# Supplementary material for: Mapping mental health complexity in esports: an exploratory psychosocial network approach
Source: BMC Psychol. 2026 Oct 19;14:1504. doi: 10.1186/s40359-026-05800-y (PMC13678908; doi:10.1186/s40359-026-05800-y)
Supplement: Supplementary file 1 [file 40359_2026_5800_MOESM1_ESM.docx]

**Supplementary Materials**

**Table S1**

*Number of participants per language version of the survey*

| Language | Count |
| --- | --- |
| *English* | 503 |
| *German* | 179 |
| *Catalan* | 127 |
| *Spanish* | 118 |
| *French* | 46 |
| *Swedish* | 32 |
| *Dutch* | 15 |
| *Icelandic* | 5 |

**Table S2**

*Number of participants per country of residence (up until 10; remaining countries grouped under “Rest”)*

| Country | Count |
| --- | --- |
| *Spain* | 217 |
| *United states of America* | 210 |
| *Germany* | 204 |
| *Sweden* | 49 |
| *France* | 46 |
| *Iceland* | 33 |
| *Netherlands* | 18 |
| *United Kingdom of Great Britain and Northern Ireland* | 17 |
| *Canada* | 16 |
| *Austria* | 12 |
| *Colombia* | 12 |
| *Poland* | 12 |
| *Switzerland* | 12 |
| *Belgium* | 11 |
| *Australia* | 10 |
| *Mexico* | 10 |
| *Rest* | 136 |

**Table S3**

*Number of participants per esports game title*

| Esports title | Count |
| --- | --- |
| *League of Legends* | 316 |
| *DOTA2* | 80 |
| *Counter-Strike* | 75 |
| *Overwatch* | 61 |
| *Rainbow Six* | 52 |
| *Valorant* | 48 |
| *Fortnite* | 44 |
| *Rocket League* | 36 |
| *EA Sports FC* | 34 |
| *Call of Duty* | 15 |
| *NBA 2K* | 10 |
| *Sim Racing* | 10 |
| *Other* | 244 |

**Table S4**

*A: Results of convergent validity analysis (affective indicators) with 95% confidence intervals*

| Construct | *1* | *2* | *3* | *4* | *5* | *6* | *7* | *8* |
| --- | --- | --- | --- | --- | --- | --- | --- | --- |
| *Anhedonia (1)* | - | - | - | - | - | - | - | - |
| *PHQ9 minus item 1 (2)* | .47 (.42-.52) | - | - | - | - | - | - | - |
| *DepressedMood (3)* | .64 (.60-.67) | .60 (.56-.64) | - | - | - | - | - | - |
| *PHQ9 minus item 2 (4)* | .50 (.45-.54) | .99 (.99-.99) | .59 (.55-.63) | - | - | - | - | - |
| *Anxiety (5)* | .51 (.47-.56) | .51 (.47-.56) | .66 (.62-.69) | .52 (.47-.56) | - | - | - | - |
| *GAD7 minus item 1 (6)* | .46 (.41-.50) | .82 (.80-.84) | .53 (.49-.57) | .81 (.79-.83) | .57 (.52-.61) | - | - | - |
| *Worrying (7)* | .51 (.47-56) | .46 (.41-.50) | .63 (.59-.67) | .46 (.41-.51) | .66 (.63-.69) | .56 (.52-.60) | - | - |
| *GAD7 minus item 2 (8* | .46 (.41-.50) | .82 (.80-.84) | .53 (.48-.57) | .82 (.80-.84) | .58 (.54-.62) | .98 (.98-.99) | .54 (.50-.59) | - |

*B: Results of convergent validity analysis (sleep indicators) with 95% confidence intervals*

| Construct | *1* | *2* | *3* | *4* |
| --- | --- | --- | --- | --- |
| *SleepQuantity (1)* | - | - | - | - |
| *SleepQuality (2)* | .70 (.67-.73) | - | - | - |
| *PSQI (3)* | .56 (.51-.64) | .60 (.55-.64) | - | - |

*Note. All correlations were significant at the 0.01 level.*

**Table S5**

*Relative Importance Network RINOutStrength (i.e.,* *sum of the weights of outgoing edges based on raw lmg/R² contributions)*

| Node | RINOutStrength | Node | RINOutStrength |
| --- | --- | --- | --- |
| DepressedMood | 0.69 | Anhedonia | 0.46 |
| Anxiety | 0.62 | EsportsStress | 0.41 |
| PhysicalFatigue | 0.60 | LackSupportEsport | 0.41 |
| SleepQuality | 0.58 | LackSupportPersonal | 0.40 |
| MentalFatigue | 0.57 | Pain | 0.38 |
| Worrying | 0.54 | LackConnection | 0.34 |
| SleepQuantity | 0.52 | LackSkill | 0.27 |
| LackAutonomy | 0.51 | GamePreoccupation | 0.26 |
| LackEmotionControl | 0.47 |  |  |

**Table S6**

*Relative Importance Network RINInStrength (i.e., sum of the weights of incoming edges based on raw lmg/R² contributions)*

| Node | RINInStrength | Node | RINInStrength |
| --- | --- | --- | --- |
| DepressedMood | 0.63 | LackEmotionControl | 0.46 |
| Anxiety | 0.59 | LackSupportEsport | 0.43 |
| PhysicalFatigue | 0.57 | EsportsStress | 0.42 |
| SleepQuality | 0.58 | LackSupportPersonal | 0.40 |
| MentalFatigue | 0.56 | Pain | 0.40 |
| SleepQuantity | 0.55 | LackConnection | 0.35 |
| Worrying | 0.54 | LackSkill | 0.30 |
| LackAutonomy | 0.49 | GamePreoccupation | 0.21 |
| Anhedonia | 0.48 |  |  |

**Table S7**

*Directed Acyclic Graph predictability (i.e., adjusted R²;* *proportion of variance in a node explained by its parents)*

| Node | AdjR² | Node | AdjR² |
| --- | --- | --- | --- |
| SleepQuality | 0.57 | LackSupportPersonal | 0.36 |
| Worrying | 0.51 | LackEmotionControl | 0.35 |
| Anxiety | 0.43 | EsportsStress | 0.30 |
| MentalFatigue | 0.43 | LackSkill | 0.27 |
| Anhedonia | 0.40 | LackConnection | 0.27 |
| PhysicalFatigue | 0.38 | LackAutonomy | 0.25 |
| Pain | 0.37 | GamePreoccupation | 0.21 |
| SleepQuantity | 0.36 | DepressedMood | 0.0000 |
| LackSupportEsport | 0.36 |  |  |

**Table S8**

*Bayesian Gaussian Graphical Copula Model partial correlations (i.e., strength of each undirected pairwise conditional dependency while controlling for all other nodes)*

| From | To | Strength | From | To | Strength |
| --- | --- | --- | --- | --- | --- |
| SleepQuantity | SleepQuality | 0.4846 | Depressed Mood | LackSupport Personal | 0.1669 |
| Anhedonia | Depressed Mood | 0.3406 | MentalFatigue | LackSkill | 0.1509 |
| Anxiety | Worrying | 0.3104 | Anxiety | Pain | 0.1504 |
| LackSupport Esports | LackSupport Personal | 0.3025 | LackEmotion Control | LackSkill | 0.1439 |
| LackSupport Esports | Lack Autonomy | 0.2819 | LackSupport Personal | LackEmotion Control | 0.1396 |
| Physical Fatigue | MentalFatigue | 0.2784 | Game Preoccupation | Lack Autonomy | 0.1365 |
| Depressed Mood | MentalFatigue | 0.2623 | EsportsStress | Game Preoccupation | 0.1273 |
| EsportsStress | Lack Autonomy | 0.2496 | Pain | Lack Connection | 0.1246 |
| Physical Fatigue | Pain | 0.2401 | SleepQuantity | MentalFatigue | 0.1178 |
| Depressed Mood | Worrying | 0.2332 | Worrying | EsportsStress | 0.1111 |
| SleepQuality | Physical Fatigue | 0.2328 | SleepQuantity | Physical Fatigue | 0.1107 |
| EsportsStress | LackSupport Esports | 0.2327 | Anxiety | MentalFatigue | 0.1068 |
| LackSkill | Lack Connection | 0.2151 | Anhedonia | Lack Connection | 0.1068 |
| LackEmotion Control | Lack Autonomy | 0.2134 | Anhedonia | LackEmotion Control | 0.0973 |
| Anxiety | LackEmotion Control | 0.1994 | Worrying | Physical Fatigue | 0.0939 |
| Lack Autonomy | Lack Connection | 0.1871 | Anhedonia | SleepQuality | 0.0851 |
| Depressed Mood | Anxiety | 0.1805 | Anxiety | SleepQuality | 0.0735 |

**Table S9**

*Directed Acyclic Graph edge-level BIC-g scores (i.e., the change in model fit score when edge is removed)*

| From | To | BIC-g | From | To | BIC-g |
| --- | --- | --- | --- | --- | --- |
| Depressed Mood | Anxiety | -286.12 | EsportsStress | LackSupport Esports | -39.12 |
| Depressed Mood | Anhedonia | -261.48 | Depressed Mood | LackEmotion Control | -37.16 |
| MentalFatigue | Physical Fatigue | -242.47 | Anxiety | Mental Fatigue | -36.29 |
| Lack Autonomy | EsportsStress | -178.70 | Lack Autonomy | Game Preoccupation | -31.71 |
| SleepQuantity | SleepQuality | -168.16 | Anxiety | Pain | -31.06 |
| LackEmotion Control | Lack Autonomy | -144.66 | Lack Autonomy | Pain | -29.33 |
| Anxiety | Worrying | -95.80 | Mental Fatigue | Sleep Quantity | -29.08 |
| LackSupport Esports | LackSupport Personal | -88.13 | Mental Fatigue | LackSkill | -28.94 |
| Depressed Mood | MentalFatigue | -85.18 | Lack Connection | LackSkill | -27.63 |
| Depressed Mood | LackSupport Personal | -78.88 | Lack Autonomy | Lack Connection | -24.86 |
| Lack Autonomy | LackSupport Esports | -77.26 | Pain | Lack Connection | -22.23 |
| Physical Fatigue | SleepQuantity | -71.96 | EsportsStress | Game Preoccupation | -21.26 |
| Depressed Mood | Worrying | -63.83 | Anxiety | SleepQuality | -16.27 |
| Physical Fatigue | Pain | -48.30 | LackEmotionControl | LackSkill | -14.40 |
| Anxiety | LackEmotion Control | -44.25 | LackSupport Esports | Lack Connection | -11.26 |
| Physical Fatigue | SleepQuality | -39.42 |  |  |  |

**Table S10**

*Directed Acyclic Graph edge-level directional probabilities (i.e., the proportion of bootstrapped networks in which, conditional on the edge being present, it pointed from node A to node B) and bootstrap adjacency frequencies (i.e., the proportion of bootstrapped networks in which an edge was present between node A and node B, irrespective of orientation)*

| From | To | Dir. | Adj. Freq. | From | To | Dir. | Adj. Freq. |
| --- | --- | --- | --- | --- | --- | --- | --- |
| Mental Fatigue | LackSkill | 0.97***^C^ | .95 | Lack Autonomy | LackSupport Esports | 0.68**^R^ | 1.00 |
| Lack Emotion Control | LackSkill | 0.93***^C^ | .94 | Anxiety | SleepQuality | 0.67**^C^ | .94 |
| Lack Autonomy | Game Preoccupation | 0.93***^R^ | .94 | Lack Autonomy | Esports Stress | 0.67**^R^ | 1.00 |
| Depressed Mood | LackSupport Personal | 0.90***^C^ | .93 | Depressed Mood | Worrying | 0.66**^R^ | 1.00 |
| Lack Autonomy | Lack Connection | 0.89***^C^ | .95 | Mental Fatigue | Sleep Quantity | 0.65**^R^ | .98 |
| Anxiety | Pain | 0.88***^C^ | .96 | Anxiety | Worrying | 0.65**^R^ | 1.00 |
| Depressed Mood | Anhedonia | 0.86***^R^ | 1.00 | Physical Fatigue | Sleep Quantity | 0.64**^R^ | .98 |
| Esports Stress | Game Preoccupation | 0.85***^R^ | .93 | Sleep Quantity | SleepQuality | 0.64**^C^ | 1.00 |
| Depressed Mood | LackEmotion Control | 0.81***^R^ | .93 | Lack Autonomy | Pain | 0.63**^C^ | .89 |
| Anxiety | LackEmotion Control | 0.80***^R^ | .99 | Mental Fatigue | Physical Fatigue | 0.61**^R^ | 1.00 |
| Physical Fatigue | Pain | 0.79**^C^ | 1.00 | Depressed Mood | Mental Fatigue | 0.58*^R^ | 1.00 |
| Pain | Lack Connection | 0.74**^C^ | .88 | Esports Stress | LackSupport Esports | 0.57*^R^ | 1.00 |
| LackSupport Esports | Lack Connection | 0.72**^C^ | .89 | Depressed Mood | Anxiety | 0.55*^R^ | 1.00 |
| Physical Fatigue | SleepQuality | 0.71**^C^ | 1.00 | Anxiety | Mental Fatigue | 0.53*^R^ | .99 |
| Lack Emotion Control | Lack Autonomy | 0.69**^R^ | .99 | Lack Connection | LackSkill | 0.52*^C^ | 1.00 |
| LackSupport Esports | LackSupport Personal | 0.68**^C^ | 1.00 |  |  |  |  |

*Note:* * weakly directed edge (< .60); ** moderately directed edge (.60 to .79); *** well directed edge (≥ .80). Superscript C connotes Compelled edges in Markov equivalence class. Superscript R connotes Reversible edges in Markov equivalence class.

**Table S11**

*Bayesian Gaussian Graphical Copula Model expected influence* *(i.e., sum of a node’s edge weights [partial correlations]; signed and absolute sums coincide as all edges were positive)*

| Node | Expected Influence | Node | Expected Influence |
| --- | --- | --- | --- |
| DepressedMood | 1.18 | EsportsStress | 0.72 |
| LackAutonomy | 1.07 | SleepQuantity | 0.71 |
| Anxiety | 1.02 | LackConnection | 0.63 |
| PhysicalFatigue | 0.96 | Anhedonia | 0.63 |
| MentalFatigue | 0.92 | LackSupportPersonal | 0.61 |
| SleepQuality | 0.88 | Pain | 0.52 |
| LackSupportEsport | 0.82 | LackSkill | 0.51 |
| LackEmotionControl | 0.79 | GamePreoccupation | 0.26 |
| Worrying | 0.75 |  |  |

**Table S12**

*Bayesian Gaussian Graphical Copula Model degree (i.e., number of connections of a node)*

| Node | Degree | Node | Degree |
| --- | --- | --- | --- |
| Anxiety | 6 | SleepQuality | 4 |
| DepressedMood | 5 | Worrying | 4 |
| LackAutonomy | 5 | LackSkill | 3 |
| LackEmotionControl | 5 | LackSupportEsport | 3 |
| MentalFatigue | 5 | LackSupportPersonal | 3 |
| PhysicalFatigue | 5 | Pain | 3 |
| Anhedonia | 4 | SleepQuantity | 3 |
| EsportsStress | 4 | GamePreoccupation | 2 |
| LackConnection | 4 |  |  |

**Table S13**

*Directed Acyclic Graph InBIC (i.e., sum of a node’s incoming edge BIC-g score)*

| Node | InBIC | Node | InBIC |
| --- | --- | --- | --- |
| Anxiety | -286.12 | LackSupportEsport | -116.38 |
| Anhedonia | -261.48 | Pain | -108.69 |
| PhysicalFatigue | -242.47 | SleepQuantity | -101.03 |
| SleepQuality | -223.85 | LackEmotionControl | -81.41 |
| EsportsStress | -178.70 | LackSkill | -70.97 |
| LackSupportPersonal | -167.01 | LackConnection | -58.35 |
| Worrying | -159.63 | GamePreoccupation | -52.97 |
| LackAutonomy | -144.66 | DepressedMood | 0.00 |
| MentalFatigue | -121.47 |  |  |

**Table S14**

*Directed Acyclic Graph OutBIC (i.e., sum of a node’s outgoing edge BIC-g score)*

| Node | OutBIC | Node | OutBIC |
| --- | --- | --- | --- |
| DepressedMood | -812.65 | LackConnection | -27.63 |
| LackAutonomy | -341.86 | Pain | -22.23 |
| MentalFatigue | -300.48 | Anhedonia | 0.00 |
| Anxiety | -223.66 | GamePreoccupation | 0.00 |
| SleepQuantity | -168.16 | LackSkill | 0.00 |
| PhysicalFatigue | -159.69 | LackSupportPersonal | 0.00 |
| LackEmotion Control | -159.07 | SleepQuality | 0.00 |
| LackSupportEsport | -99.38 | Worrying | 0.00 |
| EsportsStress | -60.38 |  |  |

**Table S15**

*Descriptive statistics (mean, standard deviation, skewness, kurtosis and range)*

| Item | M | SD | Skewness | Kurtosis | Range |
| --- | --- | --- | --- | --- | --- |
| *Anhedonia (1)* | 4.98 | 2.63 | .136 | -1.097 | 9 |
| *DepressedMood (2)* | 4.48 | 2.79 | .350 | -1.093 | 9 |
| *Anxiety (3)* | 4.58 | 2.75 | .310 | -1.097 | 9 |
| *Worrying (4)* | 4.31 | 2.83 | .413 | -1.070 | 9 |
| *EsportsStress (5)* | 3.82 | 2.72 | .707 | -.662 | 9 |
| *GamePreoccupation (6)* | 4.75 | 2.89 | .231 | -1.195 | 9 |
| *SleepQuantity (7)* | 5.52 | 2.93 | -.032 | -1.259 | 9 |
| *SleepQuality (8)* | 5.40 | 3.02 | .023 | -1.326 | 9 |
| *PhysicalFatigue (9)* | 4.96 | 2.66 | .155 | -1.075 | 9 |
| *MentalFatigue (10)* | 5.53 | 2.70 | -.116 | -1.092 | 9 |
| *Pain (11)* | 3.67 | 2.78 | .718 | -.763 | 9 |
| *LackSupportEsport (12)* | 3.61 | 2.95 | .769 | -.780 | 9 |
| *LackSupportPersonal (13)* | 4.24 | 3.04 | .436 | -1.230 | 9 |
| *LackEmotionControl (14)* | 4.24 | 2.78 | .484 | -.968 | 9 |
| *LackAutonomy (15)* | 3.60 | 2.82 | .741 | -.793 | 9 |
| *LackSkill (16)* | 5.68 | 2.82 | -.179 | -1.113 | 9 |
| *LackConnection (17)* | 4.45 | 3.18 | .379 | -1.289 | 9 |

**Table S16**

*Pearson zero-order correlations between the variables*

|  | *1* | *2* | *3* | *4* | *5* | *6* | *7* | *8* | *9* | *10* | *11* | *12* | *13* | *14* | *15* | *16* |
| --- | --- | --- | --- | --- | --- | --- | --- | --- | --- | --- | --- | --- | --- | --- | --- | --- |
| *1* | - | - | - | - | - | - | - | - | - | - | - | - | - | - | - | - |
| *2* | .64 | - | - | - | - | - | - | - | - | - | - | - | - | - | - | - |
| *3* | .51 | .66 | - | - | - | - | - | - | - | - | - | - | - | - | - | - |
| *4* | .51 | .63 | .66 | - | - | - | - | - | - | - | - | - | - | - | - | - |
| *5* | .35 | .41 | .41 | .41 | - | - | - | - | - | - | - | - | - | - | - | - |
| *6* | .33 | .36 | .34 | .33 | .39 | - | - | - | - | - | - | - | - | - | - | - |
| *7* | .33 | .39 | .38 | .35 | .24 | .31 | - | - | - | - | - | - | - | - | - | - |
| *8* | .42 | .44 | .47 | .42 | .28 | .29 | .70 | - | - | - | - | - | - | - | - | - |
| *9* | .46 | .53 | .50 | .49 | .34 | .35 | .57 | .61 | - | - | - | - | - | - | - | - |
| *10* | .51 | .62 | .57 | .52 | .36 | .31 | .51 | .52 | .617 | - | - | - | - | - | - | - |
| *11* | .38 | .46 | .49 | .46 | .40 | .30 | .32 | .37 | .500 | .41 | - | - | - | - | - | - |
| *12* | .29 | .33 | .34 | .34 | .50 | .33 | .18 | .20 | .262 | .28 | .37 | - | - | - | - | - |
| *13* | .43 | .48 | .42 | .43 | .38 | .33 | .30 | .33 | .350 | .39 | .38 | .50 | - | - | - | - |
| *14* | .46 | .53 | .54 | .47 | .44 | .38 | .34 | .35 | .387 | .43 | .42 | .40 | .45 | - | - | - |
| *15* | .38 | .42 | .39 | .38 | .55 | .41 | .27 | .28 | .326 | .31 | .42 | .55 | .46 | .50 | - | - |
| *16* | .32 | .36 | .36 | .32 | .32 | .31 | .33 | .33 | .384 | .41 | .30 | .26 | .32 | .39 | .33 | - |
| *17* | .38 | .40 | .38 | .36 | .36 | .33 | .29 | .30 | .327 | .35 | .39 | .40 | .39 | .41 | .44 | .40 |

*Note. All correlations were significant at the 0.01 level.*

**Supplementary Figure 1**
*Relative Importance Network, edge width based on normalized lmg value*

**Supplementary Figure 2**

*Bayesian Gaussian Graphical Copula model of the bespoke questionnaire data with edge labels representing partial correlations*

**Supplementary Figure 3**

*Directed Acyclic Graph of the bespoke questionnaire data – based on model-fit contribution (BIC-g scores as edge labels)*

**Supplementary Figure 4**

*Directed Acyclic Graph of the bespoke questionnaire data – based on direction probabilities (i.e., the proportion of bootstrapped networks in which, conditional on the edge being present, it pointed from node A to node B) as edge labels*
